# Pulmonary Resection for Bronchiectasis: Perioperative and Follow-Up Outcomes in a VATS-Dominant Cohort

**DOI:** 10.3390/jcm15197370

**Published:** 2026-09-23

**Authors:** Atilla Can, Hüseyin Yıldıran

**Affiliations:** Department of Thoracic Surgery, Faculty of Medicine, Selcuk University, Selçuklu, Konya 42131, Türkiye

**Keywords:** bronchiectasis, pulmonary surgical procedures, thoracic surgery, video-assisted, follow-up studies, treatment outcome

## Abstract

**Background/Objectives:** Surgical resection remains an important treatment option for selected patients with bronchiectasis who have persistent symptoms, recurrent pulmonary infections, or hemoptysis despite medical therapy. This study evaluated perioperative and follow-up outcomes after pulmonary resection for bronchiectasis in a predominantly video-assisted thoracoscopic surgery (VATS)-treated cohort. **Methods:** This retrospective single-center cohort included patients who underwent pulmonary resection for bronchiectasis between January 2009 and January 2026. Baseline and follow-up data were analyzed at the patient level, whereas operative and perioperative outcomes were assessed at the procedure level. Follow-up duration was calculated from the most recent resection to the last available clinical assessment. **Results:** Thirty-four patients underwent 37 pulmonary resections. Multilobar disease was present in 14 procedures (37.8%). VATS was used in 30 procedures (81.1%) and thoracotomy in 7 (18.9%). Median hospital stay was 11 days (range, 4–41). Postoperative complications occurred after 6 procedures (16.2%), with no 30-day mortality. Complete or partial symptomatic improvement occurred after 31 procedures (83.8%). Median follow-up was 20.8 months (interquartile range, 5.9–58.8; range, 0.4–144.2). At the last available clinical assessment, 23 patients (67.6%) had no recorded symptoms; recurrent hemoptysis occurred in 1 (2.9%) and recurrent pulmonary infection in 2 (5.9%). **Conclusions:** Pulmonary resection for bronchiectasis was associated with acceptable morbidity, no 30-day mortality, and a high rate of postoperative clinical improvement in this VATS-dominant cohort. Most patients had no recorded symptoms at their last available clinical assessment, while recurrent hemoptysis and pulmonary infection were uncommon.

## 1. Introduction

Bronchiectasis is a chronic pulmonary disease characterized by permanent dilatation of the bronchi, persistent airway inflammation, and recurrent respiratory infections, and it may occur at any age. Clinically, its most prominent manifestations include chronic cough, sputum production, and recurrent lower respiratory tract infections [1]. Impaired mucociliary clearance promotes mucus retention and bacterial persistence, perpetuating chronic inflammation and progressive airway damage that may ultimately lead to deterioration in pulmonary function [1,2]. Neutrophilic inflammation represents the predominant inflammatory phenotype, while eosinophilic or mixed inflammatory phenotypes may also occur [2,3]. Chronic productive cough and sputum production are among the most common manifestations of bronchiectasis, while dyspnea and hemoptysis occur with variable frequency across patient populations [1,4,5]. Recurrent infections and exacerbations are common and may contribute to hospitalization, impaired quality of life, and adverse clinical outcomes.

Bronchiectasis is increasingly recognized as a disease associated with substantial clinical and economic burden. Population-based studies have demonstrated increasing bronchiectasis prevalence in several countries [6,7], while recent epidemiological reviews and meta-analyses confirm a substantial overall disease burden [8,9]. Bronchiectasis is also associated with considerable healthcare utilization and mortality [6,7,8]. Patients with a frequent exacerbator phenotype experience higher rates of hospitalization, impaired quality of life, and increased mortality [10,11,12]. Coexisting chronic obstructive pulmonary disease and cardiovascular disease have been associated with poorer clinical outcomes in patients with bronchiectasis [11]. Population-based data have also shown higher frequencies of chronic obstructive pulmonary disease and previous tuberculosis among patients with bronchiectasis than among individuals without the disease [13].

The mainstay of bronchiectasis management consists of medical therapies aimed at reducing symptoms, preventing exacerbations, and limiting disease progression. However, symptoms and recurrent infections may persist despite optimal medical treatment. Surgical resection remains an important therapeutic option in carefully selected patients with localized disease that remains symptomatic despite optimized medical therapy [14,15,16]. The principal surgical objective is complete removal of diseased lung tissue to eliminate the infectious focus and achieve sustained symptom control. Complete resection has been associated with better symptomatic outcomes than incomplete resection [16]. Common indications reported in surgical series include failure of medical therapy, massive or recurrent hemoptysis, and localized destructive disease [15,16]. VATS has been successfully applied in selected patients with bronchiectasis, including contemporary series in which most anatomical resections were performed thoracoscopically [15]. Nevertheless, thoracotomy may still be required in the presence of extensive adhesions, severe inflammation, or complex anatomical changes. Overall, surgical series have reported low operative mortality and favorable symptom control in appropriately selected patients [15,16,17]. Beyond the indication for surgery, the timing of referral may also be clinically relevant. Etienne et al. reported that patients with localized bronchiectasis and recurrent symptoms despite medical treatment should be considered for surgery before the development of complications, when a more limited resection may be possible [18]. In addition, potential under-referral for surgical assessment has been described, and a structured referral framework has been proposed to help identify patients with localized, medically refractory disease who may be appropriate candidates for surgery [19].

Although the role of surgery in selected patients with bronchiectasis is well established, patient selection and the optimal timing of surgical intervention remain incompletely defined [18,19]. In addition, much of the available surgical literature consists of retrospective series accumulated over long periods, while data integrating perioperative outcomes from predominantly VATS-treated cohorts with subsequent patient-level clinical follow-up remain limited. Evaluating both early surgical outcomes and subsequent symptom control and disease recurrence may therefore provide a more complete assessment of pulmonary resection in this setting. Accordingly, this study aimed to evaluate the perioperative and follow-up outcomes of patients undergoing pulmonary resection for bronchiectasis in a VATS-dominant surgical cohort.

## 2. Materials and Methods

### 2.1. Study Design and Patients

This retrospective single-center cohort study included patients who underwent pulmonary resection for bronchiectasis at our institution between January 2009 and January 2026. The study database was closed in January 2026, corresponding to the end of the inclusion period, and no procedures performed after this cutoff were included. No age-based exclusion criterion was applied; therefore, pediatric patients who underwent pulmonary resection for bronchiectasis during the study period were included in the cohort. Medical records were reviewed to confirm that bronchiectasis was the primary indication for surgery. Duplicate records were removed, and patients whose pulmonary resection was performed primarily for an indication other than bronchiectasis were excluded. The final study cohort comprised 34 patients who underwent 37 pulmonary resections.

The study was conducted in accordance with the Declaration of Helsinki and was approved by the Selcuk University Faculty of Medicine Local Ethics Committee (approval no. 2026/168; 10 March 2026). The requirement for informed consent was waived owing to the retrospective design of the study. The manuscript was prepared in accordance with the STROBE statement for reporting observational studies.

Demographic, clinical, radiological, operative, postoperative, and follow-up data were retrospectively collected from the hospital information system, archived medical records, operative reports, and radiological imaging databases. Recorded variables included age, sex, presenting symptoms, recurrent pulmonary infections, chronic sputum production, hemoptysis, comorbidities, pulmonary function test results when available, radiological extent of disease, surgical approach, type of pulmonary resection, postoperative complications, intensive care unit (ICU) requirement, length of hospital stay, reoperation, 30-day mortality, postoperative symptomatic improvement, and follow-up outcomes.

### 2.2. Definitions and Endpoints

Bronchiectatic involvement confined to a single lobe was defined as localized disease, whereas involvement of more than one lobe was defined as multilobar disease. Hemoptysis severity was determined from the terminology documented in the medical records; because bleeding volume was not consistently quantified, a uniform volume-based threshold for massive hemoptysis could not be retrospectively applied. For operative and perioperative analyses, each pulmonary resection was treated as a separate surgical event. In patients who underwent more than one pulmonary resection, each procedure was included separately in procedure-level analyses, whereas each patient contributed only once to patient-level follow-up analyses. In these patients, follow-up duration was calculated from the most recent pulmonary resection to the last available clinical assessment.

Postoperative symptomatic improvement was retrospectively classified as complete improvement, partial improvement, or no improvement based on the wording of the clinical documentation in outpatient follow-up records; no predefined validated symptom score or prospective classification criteria were used. This outcome was analyzed at the procedure level. Symptom status at the last available clinical assessment was evaluated separately at the patient level. Follow-up outcomes included symptoms documented at the last available clinical assessment, recurrent hemoptysis, recurrent pulmonary infection, and unplanned reoperation for postoperative complications. Planned or staged contralateral pulmonary resections were considered separate surgical procedures and were not classified as unplanned reoperations. Thirty-day mortality was defined as death occurring within 30 days after surgery.

The study outcomes included postoperative complications, length of hospital stay, ICU requirement, 30-day mortality, postoperative symptomatic improvement, and patient-level clinical outcomes during follow-up.

### 2.3. Surgical Management

Patients were considered for pulmonary resection primarily because of recurrent pulmonary infections and/or hemoptysis in the presence of anatomically resectable bronchiectatic disease. In patients presenting with massive hemoptysis, pulmonary resection was performed after control of the acute bleeding episode as definitive treatment to reduce the risk of recurrent potentially life-threatening hemorrhage. The operative strategy aimed to achieve complete anatomical resection of the radiologically identified diseased lung while preserving uninvolved pulmonary parenchyma; intentionally incomplete resection of the targeted bronchiectatic segments or lobes was not performed.

The surgical approach was selected according to disease extent and localization, radiological findings, the presence of pleural adhesions, hilar anatomy, and surgeon preference. VATS was preferred when anatomical and operative conditions were considered suitable, whereas thoracotomy was used in patients with extensive adhesions, severe inflammatory changes, or complex hilar anatomy. These conditions were considered relative limitations to a VATS approach because they could make safe hilar dissection and vascular or bronchial control more challenging. The extent of pulmonary resection was determined according to the localization and distribution of bronchiectatic involvement and included lobectomy, bilobectomy, segmentectomy, and lingulectomy, as appropriate.

### 2.4. Statistical Analysis

Statistical analyses were performed using IBM SPSS Statistics for Windows, version 25.0 (IBM Corp., Armonk, NY, USA). Analyses were primarily descriptive because of the limited cohort size and the small number of postoperative events. Patient-level variables, including demographic characteristics and follow-up outcomes, were analyzed using the number of unique patients as the denominator, whereas operative and perioperative variables were analyzed at the procedure level. Continuous variables were summarized as mean ± standard deviation or median with interquartile range (IQR) and range, as appropriate, and categorical variables were presented as numbers and percentages. Missing data were analyzed on an available-case basis and were not imputed. Because postoperative complications occurred after only six procedures, inferential analyses aimed at identifying factors associated with postoperative complications, including correlation analyses, logistic regression, or multivariable modeling, were not performed. Given the limited number of events, such analyses were considered statistically unstable and at substantial risk of producing unreliable or spurious associations.

## 3. Results

### 3.1. Patient Characteristics

During the study period, 34 patients underwent 37 pulmonary resections for bronchiectasis. Three patients underwent two separate pulmonary resections. Patient-level demographic and baseline clinical characteristics were assessed at the time of the first operation. The mean age at first operation was 40.8 ± 18.2 years, and the median age was 35.5 years (range, 4–82 years). Nineteen patients (55.9%) were female and 15 (44.1%) were male. Two patients (5.9%) were younger than 18 years at the time of their first operation.

Recurrent pulmonary infections were documented in 21 patients (61.8%), chronic sputum production in 23 (67.6%), and hemoptysis in 6 (17.6%). Recurrent pulmonary infection and hemoptysis represented the principal clinical indications for considering surgical resection in this cohort. Among the 32 patients with available data regarding hemoptysis severity, massive hemoptysis was documented in 2 (6.3%). Because the indication for surgery was not retrospectively coded as a single mutually exclusive variable, indication-specific frequencies beyond these documented clinical features were not analyzed separately. Symptom duration was incompletely documented and was therefore not analyzed further. Paired preoperative and postoperative spirometric measurements, including forced expiratory volume in 1 s (FEV1) and forced vital capacity (FVC), were not available in a sufficiently complete and standardized manner to permit a meaningful longitudinal analysis. Patient-level demographic and clinical characteristics are summarized in Table 1.

### 3.2. Radiological and Operative Findings

Of the 37 pulmonary resections, 23 (62.2%) were performed for disease confined to a single lobe, whereas multilobar involvement was present in 14 (37.8%). Seventeen procedures (45.9%) involved the right hemithorax and 20 (54.1%) involved the left hemithorax.

VATS was used in 30 of 37 procedures (81.1%), whereas thoracotomy was performed in 7 (18.9%). Lobectomy was the predominant resection type, accounting for 32 procedures (86.5%); selected lobectomies were combined with limited wedge resection when required. Additional resection types included bilobectomy, lingulectomy, and segmentectomy. Operative characteristics are summarized in Table 2.

### 3.3. Postoperative Outcomes

The median length of hospital stay was 11 days (range, 4–41 days). Postoperative ICU admission was required after one procedure (2.7%), and no 30-day mortality occurred.

At least one postoperative complication occurred after 6 of 37 procedures (16.2%). Complications included prolonged air leak after four procedures, empyema after two, and chylous drainage after one; more than one complication could occur after the same procedure. Two procedures required unplanned reoperation for postoperative complications.

Complete postoperative symptomatic improvement was documented after 26 procedures (70.3%), partial improvement after 5 (13.5%), and no improvement after 6 (16.2%). Overall clinical improvement, defined as complete or partial symptomatic improvement, was observed after 31 of 37 procedures (83.8%).

### 3.4. Follow-Up Outcomes

Follow-up data were available for all 34 patients. The median follow-up duration from the most recent pulmonary resection to the last available clinical assessment was 20.8 months (IQR, 5.9–58.8 months; range, 0.4–144.2 months). At the last available clinical assessment, 23 patients (67.6%) had no recorded symptoms. Among the remaining patients, dyspnea was documented in 4 (11.8%), chest pain in 4 (11.8%), and cough in 3 (8.8%).

During follow-up, recurrent hemoptysis was documented in 1 patient (2.9%) and recurrent pulmonary infection in 2 (5.9%). Two patients (5.9%) underwent unplanned reoperation for postoperative complications. Planned or staged contralateral pulmonary resections were not classified as unplanned reoperations. Patient-level follow-up outcomes are summarized in Table 3.

## 4. Discussion

The primary goals of bronchiectasis surgery are to achieve sustained symptom control, prevent recurrent complications, and improve quality of life. Large surgical series have reported high rates of symptomatic improvement, low mortality, and acceptable morbidity in appropriately selected patients. Across these surgical series, more than 90% of patients were reported to become asymptomatic or experience clinical improvement after surgery [20,21,22]. A meta-analysis reported pooled rates of 66.5% for becoming asymptomatic, 27.5% for clinical improvement, and 9.1% for no clinical improvement after surgery [23]. In our cohort, overall clinical improvement was observed after 83.8% of procedures, consistent with these previous reports. Across the cited surgical series, operative mortality ranged from 0% to 1.7% and postoperative morbidity from 8.8% to 23.0% [16,20,21,22,24]; the meta-analysis reported pooled mortality and morbidity rates of 1.5% and 16.7%, respectively [23]. In the present study, no 30-day mortality occurred, and postoperative complications were observed after 16.2% of procedures. Prolonged air leak was the most frequent complication, followed by empyema and chylous drainage. Complete resection has consistently been associated with better symptom control and lower rates of persistent symptoms or unfavorable outcomes [20,21]. Conversely, incomplete resection has been identified as a risk factor for persistent symptoms and poorer clinical outcomes in several studies [20,21,25,26]. In our surgical practice, the operative objective was complete anatomical removal of the radiologically identified bronchiectatic parenchyma selected for resection, and intentionally incomplete resection was not performed. However, completeness of resection was not recorded as a separate study variable; therefore, a formal complete-versus-incomplete resection analysis could not be performed.

VATS has become increasingly adopted for pulmonary resections in patients with bronchiectasis. Contemporary adult data support the feasibility of VATS in carefully selected patients with bronchiectasis-destroyed lung. In the series by Xu et al., 9 of 64 patients (14.1%) scheduled for VATS required conversion to thoracotomy because of dense pleural adhesions or a frozen hilum, emphasizing the importance of careful patient selection and assessment of technical feasibility [27]. In pediatric bronchiectasis, VATS has also been associated with less blood loss, shorter duration of ventilatory support, less postoperative pain, and shorter hospitalization compared with open surgery [28]. In our cohort, VATS was used in 30 of 37 procedures (81.1%), indicating that a minimally invasive approach was feasible in the majority of selected patients. The predominance of VATS in this cohort also provides real-world outcome data on the application of minimally invasive thoracic surgery to a non-malignant chronic pulmonary disease. However, the present study was not designed or powered to compare VATS with thoracotomy; therefore, no conclusions regarding the relative superiority of either approach can be drawn from our data. Thoracotomy may still be required in patients with extensive adhesions, severe inflammatory changes, or complex hilar anatomy. The median postoperative hospital stay of 11 days in our cohort was longer than that typically expected in contemporary VATS practice. This finding may partly reflect the long inclusion period and cautious postoperative management, including decisions regarding chest tube removal and discharge, after resection of chronically inflamed bronchiectatic lung. However, the specific determinants of length of stay were not systematically recorded, and a causal explanation cannot be established from the present data.

Surgical outcomes in bronchiectasis may be influenced by several factors, including disease extent, symptom duration, and the ability to achieve complete resection. In a pediatric series, prolonged symptom duration was associated with an increased risk of postoperative complications [29]. Prolonged preoperative symptom duration has also been reported in adult surgical series, with a mean duration of approximately 43 months in one study [20] and a mean duration of 4 years in another [22]. In our cohort, however, symptom duration was incompletely documented and therefore could not be reliably evaluated in relation to postoperative outcomes. Multilobar disease was present in 14 of 37 procedures (37.8%). Previous studies have reported better long-term outcomes in patients with localized bronchiectasis, whereas more extensive disease has been associated with less favorable long-term outcomes [20,21,30]. Because only six postoperative complications occurred in our cohort, the study was not sufficiently powered to evaluate associations between disease extent, symptom duration, or other clinical factors and postoperative morbidity. These variables should therefore be interpreted as descriptive characteristics of the cohort rather than predictors of surgical outcome.

Pulmonary resection for bronchiectasis has also been reported to provide favorable outcomes in appropriately selected pediatric patients. In a series of 176 children, operative mortality and morbidity rates were 0% and 13%, respectively; 73.3% of patients achieved an excellent outcome and 23.3% showed clinical improvement [31]. In the same study, 96.6% of patients who underwent complete resection benefited from surgery, whereas incomplete resection was considered primarily a palliative option in selected circumstances [31]. In our cohort, only two patients were younger than 18 years at the time of their first operation, and no 30-day mortality occurred in either patient. Given the very small number of pediatric patients, no age-specific conclusions can be drawn from the present series. Accordingly, the findings of the present study should be interpreted primarily as representative of an adult surgical population.

From a clinical perspective, our findings support maintaining pulmonary resection as a treatment option for carefully selected patients with anatomically resectable bronchiectasis who continue to experience recurrent pulmonary infections, persistent symptoms, or hemoptysis despite appropriate medical management. In patients presenting with massive hemoptysis, surgery in our cohort was not performed as an emergency intervention for uncontrolled active bleeding; rather, resection was undertaken after stabilization as definitive treatment intended to reduce the risk of recurrent potentially life-threatening hemorrhage. Given the heterogeneity of bronchiectasis, decisions regarding surgical treatment should be individualized and ideally made through multidisciplinary assessment, taking into account disease distribution, persistent symptom burden, and the feasibility of complete resection while preserving uninvolved lung parenchyma. Future prospective multicenter studies incorporating standardized pulmonary function testing, validated quality-of-life measures, microbiological profiles, and longitudinal radiological assessment are needed to better define patient selection, optimal timing of surgery, and the long-term outcomes of minimally invasive pulmonary resection.

### Limitations

This study has several limitations. First, its retrospective, single-center design and relatively small sample size may limit the generalizability of the findings. The small number of postoperative complications also limited the statistical power to explore associations between clinical or disease-related characteristics and postoperative morbidity. Accordingly, correlation, regression, and other predictor analyses were not performed because the resulting estimates would have been unstable and potentially misleading. In addition, symptom duration, serial pulmonary function measurements, microbiological culture data, and postoperative radiological assessments were not consistently available in a standardized form in the retrospective records and therefore could not be analyzed systematically. Information regarding preoperative bronchial artery embolization was also not consistently documented and therefore could not be analyzed systematically. Follow-up relied on routine clinical documentation rather than standardized prospective assessments; consequently, changes in pulmonary function, validated quality-of-life or functional outcomes, pathogen-specific associations with recurrent infection, and serial radiological changes could not be evaluated. Furthermore, follow-up duration varied substantially among patients. The long inclusion period may also have introduced temporal heterogeneity, as surgical techniques, perioperative care, patient selection, and the adoption of VATS evolved over time. Because the cohort size was limited, outcomes could not be meaningfully stratified by treatment era, and this potential time-related effect should be considered when interpreting the findings.

Despite these limitations, the study has several strengths. It reflects real-world surgical practice over a long study period, includes a predominantly VATS-treated cohort, and provides both procedure-level perioperative outcomes and patient-level follow-up data. Follow-up information was available for all patients, allowing assessment of subsequent symptom status, recurrent hemoptysis, and recurrent pulmonary infection beyond the initial postoperative hospitalization.

## 5. Conclusions

Pulmonary resection for bronchiectasis was associated with favorable perioperative and follow-up outcomes in this VATS-dominant cohort. Postoperative morbidity was acceptable, no 30-day mortality occurred, and most procedures resulted in complete or partial symptomatic improvement. At follow-up, most patients had no recorded symptoms at their last available clinical assessment, while recurrent hemoptysis and pulmonary infection were uncommon. These findings support the role of pulmonary resection in carefully selected patients with bronchiectasis.

## Figures and Tables

**Table 1:** Patient-level demographic and clinical characteristics.

Variable	Value
Number of patients	34
Age at first pulmonary resection, years, mean ± SD	40.8 ± 18.2
Age at first pulmonary resection, years, median (range)	35.5 (4–82)
Female sex	19 (55.9)
Male sex	15 (44.1)
Pediatric patients (<18 years)	2 (5.9)
Chronic sputum production	23 (67.6)
Recurrent pulmonary infections	21 (61.8)
Hemoptysis	6 (17.6)
Massive hemoptysis ^a^	2 (6.3)

Values are presented as n (%) unless otherwise stated. SD, standard deviation. ^a^ Data on hemoptysis severity were available for 32 patients; massive hemoptysis was documented in 2 of these patients (6.3%).

**Table 2:** Operative characteristics and perioperative outcomes.

Variable	Value
Number of surgical procedures	37
Disease extent	
Localized disease	23 (62.2)
Multilobar disease	14 (37.8)
Side of resection	
Right	17 (45.9)
Left	20 (54.1)
Surgical approach	
VATS	30 (81.1)
Thoracotomy	7 (18.9)
Resection type	
Lobectomy ^a^	32 (86.5)
Bilobectomy	2 (5.4)
Lingulectomy	2 (5.4)
Segmentectomy	1 (2.7)
Length of hospital stay, days, median (range)	11 (4–41)
Postoperative ICU admission	1 (2.7)
Any postoperative complication ^b^	6 (16.2)
Prolonged air leak	4 (10.8)
Empyema	2 (5.4)
Chylous drainage	1 (2.7)
Unplanned reoperation for postoperative complications	2 (5.4)
30-day mortality	0 (0)
Complete symptomatic improvement	26 (70.3)
Partial symptomatic improvement	5 (13.5)
No symptomatic improvement	6 (16.2)
Overall clinical improvement ^c^	31 (83.8)

Values are presented as n (%) unless otherwise stated. VATS, video-assisted thoracoscopic surgery; ICU, intensive care unit. ^a^ The lobectomy category includes lobectomy combined with limited wedge resection. ^b^ More than one postoperative complication could occur after the same procedure. ^c^ Overall clinical improvement was defined as complete or partial symptomatic improvement.

**Table 3:** Patient-level follow-up outcomes.

Variable	Value
Patients with available follow-up data	34 (100)
Follow-up duration, months, median (IQR; range)	20.8 (5.9–58.8; 0.4–144.2)
**Symptoms at last available clinical assessment**	
No recorded symptoms	23 (67.6)
Dyspnea	4 (11.8)
Chest pain	4 (11.8)
Cough	3 (8.8)
**Events during follow-up**	
Recurrent hemoptysis	1 (2.9)
Recurrent pulmonary infection	2 (5.9)
Unplanned reoperation for postoperative complications	2 (5.9)

Values are presented as n (%) unless otherwise stated. Follow-up duration was calculated from the most recent pulmonary resection to the last available clinical assessment. Planned or staged contralateral pulmonary resections were not classified as unplanned reoperations. IQR, interquartile range. The bolded parts serve as category headings.

## Data Availability

The data presented in this study are available from the corresponding author upon reasonable request. The data are not publicly available due to patient privacy and ethical restrictions.
